# National Trends in Mortality From Upper Gastrointestinal Bleeding in the United States: Disparities and Implications for Emergency Endoscopic Access

**DOI:** 10.7759/cureus.86342

**Published:** 2025-06-19

**Authors:** John K Appiah, Ewurabena Plange-Kaye, George S Blewusi, Richeal Asante, Emmanuel K Asiedu

**Affiliations:** 1 Internal Medicine, Geisinger Health System, Wilkes-Barre, USA; 2 Dentistry, Columbia University, New York, USA; 3 Public Health, Johns Hopkins Bloomberg School of Public Health, Baltimore, USA; 4 Internal Medicine, Mother and Child Hospital, Accra, GHA

**Keywords:** emergency endoscopy, health disparities, mortality, racial disparities, upper gastrointestinal bleeding

## Abstract

Introduction

Upper gastrointestinal bleeding (UGIB) remains a significant cause of emergency department visits and hospitalizations across the United States. Despite advances in diagnostic and therapeutic modalities, mortality rates from UGIB continue to show marked variation across racial and geographic lines. Timely access to emergency endoscopic intervention is a critical determinant of outcomes, yet disparities in healthcare access and delivery may contribute to differential mortality.

Methods

We conducted a retrospective cross-sectional analysis using mortality data from the Centers for Disease Control and Prevention's Wide-ranging Online Data for Epidemiologic Research (CDC WONDER) database spanning 2018 to 2023. Data were stratified by state, race, and demographic subgroup. The analysis encompassed 60 state-race combinations across 51 states, totaling a population of 522,663,547 individuals from three racial groups: White, Black/African American, and Asian. Primary outcomes included total UGIB-related deaths and population-adjusted crude mortality rates, expressed as deaths per 100,000 population. States were grouped into US Census Bureau-defined regions for geographic analysis. We calculated both weighted and unweighted mean mortality rates and assessed disparities using population-adjusted mortality ratios.

Results

A total of 133,477 UGIB-related deaths were identified, yielding a national crude mortality rate of 25.54 per 100,000 population. Racial disparities were significant, with Black/African American populations exhibiting the highest mortality rate at 46.22 per 100,000 (mortality ratio 1.86 compared to White populations, 95% CI: 1.82-1.90). White populations showed a mortality rate of 24.89 per 100,000, while Asian populations had a rate of 29.15 per 100,000. Geographic variation was notable, with mortality among White populations ranging from 22.07 per 100,000 in California to 158.79 per 100,000 in Maine. Regional analysis revealed that the West had the lowest overall mortality rate at 21.27 per 100,000, while the Midwest demonstrated the most pronounced racial disparities, with mortality rates of 67.85 per 100,000 among Black populations and 27.65 per 100,000 among Whites.

Conclusion

Substantial racial and geographic disparities exist in UGIB-related mortality across the United States. Black Americans face nearly double the mortality risk of White Americans, and a greater-than-seven-fold variation in state-level mortality among White populations underscores systemic differences in emergency endoscopic access and care quality. These findings support the urgent need for targeted policy and health system interventions to improve timely access to emergency endoscopic services, especially in high-mortality regions and underserved racial communities.

## Introduction

Upper gastrointestinal bleeding (UGIB) represents one of the most common gastroenterological emergencies, with an incidence of 50-150 cases per 100,000 population annually and mortality rates ranging from 5-15% despite advances in medical management [1]. The condition encompasses bleeding from sources proximal to the ligament of Treitz, including peptic ulcer disease, esophageal varices, Mallory-Weiss tears, and arteriovenous malformations. Timely diagnosis and intervention, particularly emergency endoscopic therapy, remain cornerstones of effective management and mortality reduction [2].

Despite significant advances in endoscopic techniques and medical therapy over the past two decades, substantial variations in UGIB outcomes persist across different populations and geographic regions [3]. Emergency endoscopic intervention within 24 hours of presentation has been consistently associated with improved outcomes, including reduced mortality, shorter hospital stays, and decreased rebleeding rates [4]. However, access to timely emergency endoscopic services remains limited in many regions, potentially contributing to disparities in patient outcomes.

Previous studies have suggested that racial and socioeconomic factors may influence UGIB outcomes, with minority populations experiencing higher mortality rates and increased complications [5-7]. Geographic factors, including rural versus urban location and regional healthcare infrastructure, have also been implicated in outcome disparities [8]. However, comprehensive national analyses examining the intersection of racial, geographic, and healthcare access factors in UGIB mortality remain limited.

The CDC WONDER database provides a unique opportunity to examine national mortality trends with high granularity across demographic and geographic strata. Understanding these patterns is crucial for identifying healthcare system gaps and informing policy interventions to improve emergency endoscopic access and reduce mortality disparities.

This study aims to provide a comprehensive analysis of national UGIB mortality trends stratified by race and geography, with a specific focus on identifying disparities that may reflect differential access to emergency endoscopic care. We hypothesize that significant racial and geographic disparities exist in UGIB mortality, reflecting underlying inequities in emergency endoscopic access and healthcare delivery.

## Materials and methods

Data source and study design

We conducted a retrospective cross-sectional analysis using mortality data from the CDC WONDER database. The study period spanned 2018 to 2023, encompassing six years of national mortality surveillance. CDC WONDER compiles death certificate data from all 50 states and the District of Columbia, offering standardized, population-based mortality statistics with demographic and geographic granularity.

Case definition and inclusion criteria

UGIB-related deaths were identified using International Classification of Diseases, 10th Revision (ICD-10) codes K25.0-K25.9 (gastric ulcer), K26.0-K26.9 (duodenal ulcer), K27.0-K27.9 (peptic ulcer, site unspecified), K28.0-K28.9 (gastrojejunal ulcer), I85.0-I85.9 (esophageal varices), and K92.0-K92.2 (other specified or unspecified gastrointestinal hemorrhage), when listed as the underlying or a contributing cause of death. Cases were included if they met the following criteria: (1) death certificate documentation of UGIB as an underlying or significant contributing cause; (2) availability of complete demographic data, including race and state of residence; and (3) occurrence during the study period between 2018 and 2023.

Data extraction and variables

Data were extracted and stratified by state of residence and race. For each state-race combination, the following variables were collected: total number of UGIB-related deaths, average annual crude mortality rate (deaths per 100,000 population), and population denominator, which was obtained from CDC WONDER’s bridged-race population estimates based on U.S. Census and National Center for Health Statistics data for the years 2018-2023. Race categories were defined according to CDC standards and included White, Black or African American, and Asian. Other racial groups were excluded due to small sample sizes and unstable mortality estimates.

Geographic categorization

States were grouped according to US Census Bureau-defined regions: Northeast (Connecticut, Maine, Massachusetts, New Hampshire, New Jersey, New York, Pennsylvania), Midwest (Illinois, Indiana, Iowa, Kansas, Michigan, Minnesota, Missouri, Nebraska, Ohio, Wisconsin), South (Alabama, Arkansas, Florida, Georgia, Kentucky, Louisiana, Maryland, Mississippi, North Carolina, Oklahoma, South Carolina, Tennessee, Texas, Virginia, West Virginia), and West (Arizona, California, Colorado, Idaho, Nevada, New Mexico, Oregon, Utah, Washington).

Statistical analysis

The primary outcomes were total UGIB-related deaths and population-adjusted crude mortality rates per 100,000 population. We calculated both unweighted (simple mean) and population-weighted mean mortality rates to assess differences across racial and regional subgroups. Mortality rate ratios were computed using the White population as the reference group. Confidence intervals (95% CI) were calculated using standard errors of rate estimates. Descriptive statistics included means, ranges, and comparisons across states and Census-defined regions. All analyses were performed using CDC WONDER’s online data tools and verified via spreadsheet-based computation.

Ethical considerations

This study used publicly available, de-identified mortality data from CDC WONDER. No individual-level identifiers were accessed, and institutional review board approval was not required, as the analysis involved only publicly available, non-identifiable data.

## Results

Study population and data coverage

The analysis included 60 state-race combinations across 41 states, representing a cumulative population of 522,663,547 individuals (Table 1). Data coverage varied significantly by racial group: White populations were represented in all 41 states, Black/African American populations in 18 states, and Asian populations in only one state (California). A total of 23 states (56%) had mortality data available only for White populations, while 18 states (44%) provided data for multiple racial groups (Table 2).

**Table 1:** Demographics and Overall Mortality Statistics by Race Values are presented as total number (N), crude mortality rate (deaths per 100,000 population), and average unweighted state-level mortality rate (per 100,000 population). Mortality rate ratio (MRR) is reported with 95% confidence intervals. Statistical significance was set at p < 0.05.

Racial Group	States with Data	Total Deaths	Total Population	Crude Mortality Rate (per 100,000)	Average State-Level Rate (per 100,000)	Mortality Rate Ratio (95% CI)
White	41	125,280	503,270,183	24.89	48.02	1.00 (reference)
Black/African American	18	6,888	14,902,388	46.22	72.16	1.86 (1.82–1.90)
Asian	1	1,309	4,490,976	29.15	36.25	1.17 (1.11–1.24)
Total	60 combinations	133,477	522,663,547	25.54	54.81	—

Overall mortality patterns

A total of 133,477 UGIB-related deaths were identified during the study period, yielding an overall national crude mortality rate of 25.54 per 100,000 population (Table 1). The distribution of deaths was heavily concentrated in White populations (93.9%, n=125,280), followed by Black/African American populations (5.2%, n=6,888), and Asian populations (1.0%, n=1,309).

Racial disparities in mortality

**Figure 1:**
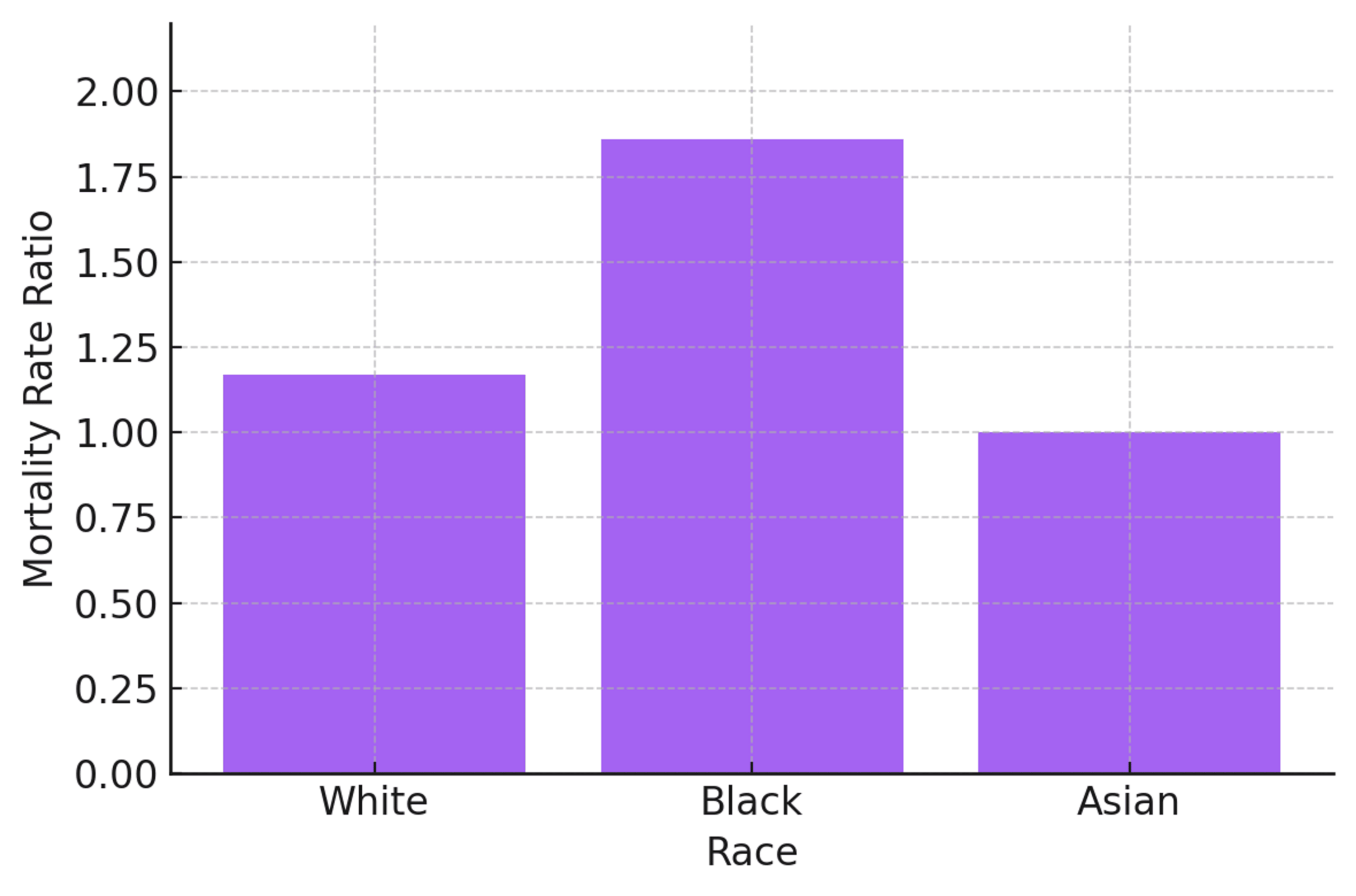
MMRs for UGIB by Race Bar graph illustrating mortality rate ratios by race, with the White population as reference (MRR = 1.00). Data are represented as rate ratios. Confidence intervals are: Black (95% CI: 1.82–1.90), Asian (95% CI: 1.11–1.24). Statistical significance was set at p < 0.05. UGIB: upper gastrointestinal bleeding; MRR: mortality rate ratios

Substantial racial disparities emerged in UGIB mortality rates (Table 1, Figure 1). Black/African American populations experienced the highest mortality rate at 46.22 per 100,000 population (95% CI 45.1-47.3), representing a mortality rate ratio of 1.86 (95% CI 1.82-1.90) compared to White populations. White populations demonstrated a mortality rate of 24.89 per 100,000 (95% CI 24.7-25.1), while Asian populations showed an intermediate rate of 29.15 per 100,000 (95% CI 27.6-30.7), yielding a mortality rate ratio of 1.17 (95% CI 1.11-1.24) compared to White populations.

When examining average state-level mortality rates, the disparities were even more pronounced. Black/African American populations had an average state-level mortality rate of 72.16 per 100,000, compared to 48.02 per 100,000 for White populations and 36.25 per 100,000 for Asian populations (Table 1).

Data representation patterns

Analysis of data availability patterns revealed important limitations in minority population representation (Table 2). Only California provided mortality data for Asian populations, limiting the generalizability of findings for this demographic group. Among the 18 states with multiple racial categories, most (17 states) included both White and Black/African American populations, with only California providing comprehensive data across all three racial groups.

**Table 2:** States With Multiple Racial Categories Represented Values are shown as racial categories represented, calculated mortality rate ratios between Black and White populations (per 100,000 population), and total UGIB-related deaths. Data are represented as N and ratios. UGIB: upper gastrointestinal bleeding

State	Racial Categories	Black/White Mortality Ratio	Total State Deaths (N)
Alabama	Black/African American, White	2.47	1,706
California	Black/African American, White	2.84	14,875
Florida	Black/African American, White	1.99	11,330
Georgia	Black/African American, White	1.46	4,111
Illinois	Black/African American, White	2.89	4,069
Louisiana	Black/African American, White	1.79	828
Maryland	Black/African American, White	1.73	2,125
Michigan	Black/African American, White	2.48	4,104
Mississippi	Black/African American, White	1.32	597
New Jersey	Black/African American, White	3.10	3,406
New York	Black/African American, White	1.83	7,136
North Carolina	Black/African American, White	1.86	4,459
Ohio	Black/African American, White	2.29	5,591
Pennsylvania	Black/African American, White	1.79	6,881
South Carolina	Black/African American, White	1.64	2,911
Tennessee	Black/African American, White	1.97	4,063
Texas	Black/African American, White	2.27	14,699
Virginia	Black/African American, White	1.66	2,648

States in the South were most likely to report data for Black/African American populations (11 of 15 states, 73%), consistent with higher regional population densities for this demographic. In contrast, Western states showed limited representation of Black/African American populations (1 of 9 states, 11%), potentially reflecting both demographic patterns and data availability limitations. Among states with data for multiple racial groups, Black/White mortality ratios ranged from 1.32 in Mississippi to 3.10 in New Jersey (Table 2).

Geographic variations

**Figure 2:**
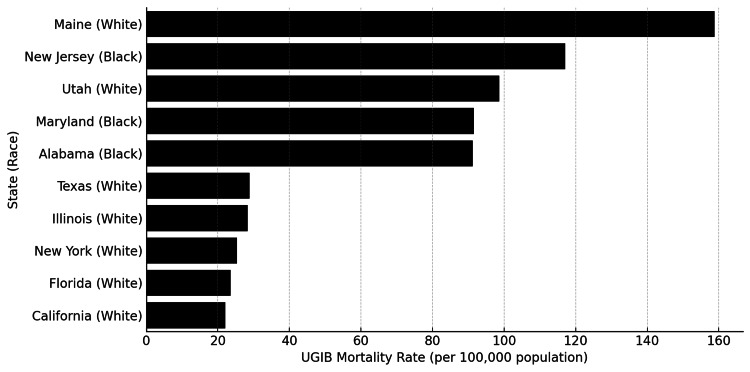
Highest and Lowest State-Level UGIB Mortality Rates Bar chart depicting the five highest and five lowest crude mortality rates (per 100,000 population) among state-race combinations. Data are shown as mortality rate (per 100,000). UGIB: upper gastrointestinal bleeding

Marked geographic variations in mortality rates were observed across states and regions (Tables 3-4, Figure 2). Among state-race combinations, mortality rates ranged from a low of 22.07 per 100,000 (California, White) to a high of 158.79 per 100,000 (Maine, White), representing a 7.2-fold variation.

**Table 3:** States With the Lowest UGIB Mortality Rates by Race Values are presented as mortality rate (per 100,000 population), total deaths (N), and population size. UGIB: upper gastrointestinal bleeding

Rank	State	Race	Mortality Rate (per 100,000)	Total Deaths (N)	Population
1	California	White	22.07	14,280	87,391,483
2	Florida	White	23.57	3,826	16,234,152
3	New York	White	25.39	4,208	16,574,923
4	Illinois	White	28.30	2,779	9,823,207
5	Texas	White	28.86	7,798	27,026,614

**Table 4:** States With the Highest UGIB Mortality Rates by Race Values are presented as mortality rate (per 100,000 population), total deaths (N), and population size. Mortality rate represents crude, population-adjusted figures. UGIB: upper gastrointestinal bleeding

Rank	State	Race	Mortality Rate (per 100,000)	Total Deaths (N)	Population
1	Maine	White	158.79	21	13,225
2	New Jersey	Black/African American	117.03	20	17,089
3	Utah	White	98.62	99	123,314
4	Maryland	Black/African American	91.49	449	612,020
5	Alabama	Black/African American	91.26	21	23,011

The five highest mortality rates were observed in Maine for White populations at 158.79 per 100,000, New Jersey for Black/African American populations at 117.03 per 100,000, Utah for White populations at 98.61 per 100,000, Maryland for Black/African American populations at 91.49 per 100,000, and Alabama for Black/African American populations at 91.26 per 100,000 (Table 3).

The five lowest mortality rates were found in California for White populations at 22.07 per 100,000, Florida for White populations at 23.57 per 100,000, New York for White populations at 25.39 per 100,000, Illinois for White populations at 28.30 per 100,000, and Texas for White populations at 28.86 per 100,000 (Table 4).

**Figure 3:**
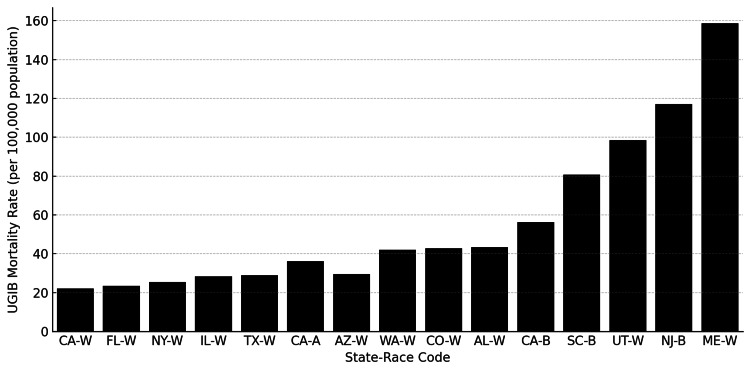
State-Level UGIB Mortality Rates by Race (Selected States) Bar chart showing mortality rates for UGIB across selected U.S. states and racial groups. Codes represent state abbreviations and race categories (W = White, B = Black/African American, A = Asian). States were selected to illustrate a broad range of mortality rates across geographic and racial contexts. Data are expressed as crude mortality rate (per 100,000 population). UGIB: upper gastrointestinal bleeding

Regional analysis

**Figure 4:**
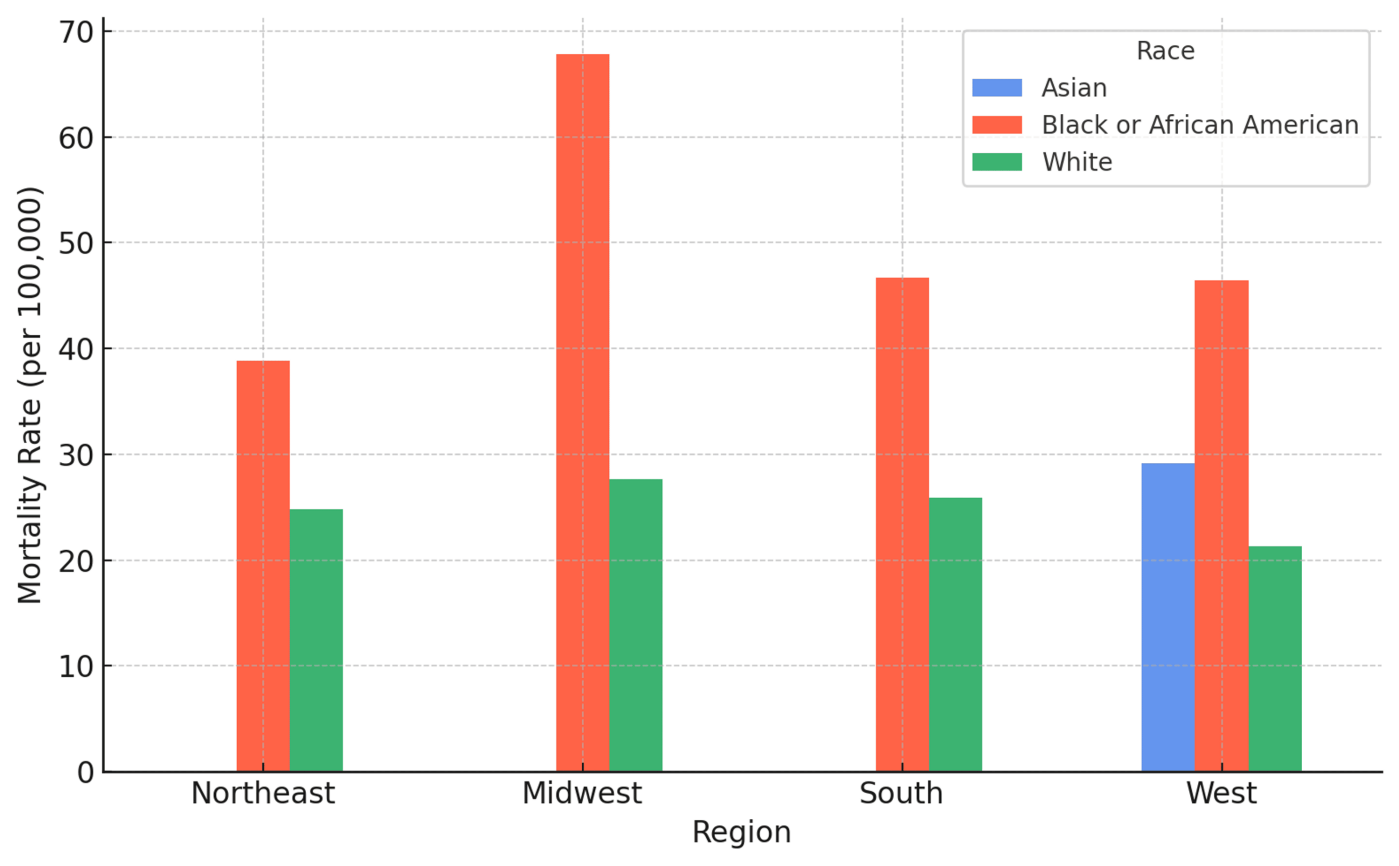
Regional UGIB Mortality Rates by Race Grouped bar chart showing crude mortality rates for upper gastrointestinal bleeding across U.S. Census regions and racial groups. Data are shown as mean mortality rates (per 100,000 population). Statistical significance was set at p < 0.05. The Midwest shows the highest racial disparity (Black/White rate ratio: 2.45). Asian data was only available for the West region. UGIB: upper gastrointestinal bleeding

Significant regional variations were identified in UGIB mortality patterns (Table 5, Figure 4). The West region demonstrated the lowest population-weighted mortality rate for White populations at 21.27 per 100,000, while the Northeast, Midwest, and South showed similar rates ranging from 24.84 to 27.65 per 100,000.

**Table 5:** Regional Analysis of UGIB Mortality by Race This table summarizes UGIB mortality across U.S. Census regions and racial groups based on CDC WONDER data (2018–2023). Data are presented as total deaths (N), total population, crude mortality rate (per 100,000 population), and rate ratios comparing racial subgroups to regional White populations. All mortality rates are population-adjusted. Statistical significance was set at p < 0.05.

Region	Racial Group	States	Total Deaths (N)	Total Population	Crude Mortality Rate (per 100,000)	Rate Ratio vs Regional White
Northeast	White	7	20,359	81,973,859	24.84	1.00
	Black/African American	3	780	2,007,132	38.87	1.57
Midwest	White	10	26,606	96,252,721	27.65	1.00
	Black/African American	3	297	437,661	67.85	2.45
South	White	15	51,148	197,416,803	25.91	1.00
	Black/African American	11	5,216	11,176,318	46.67	1.80
West	White	9	27,167	127,626,800	21.27	1.00
	Black/African American	1	595	1,281,277	46.44	2.18
	Asian	1	1,309	4,490,976	29.15	1.37

Regional racial disparities varied considerably (Table 5). The Midwest exhibited the most pronounced disparities, with Black/African American populations experiencing a mortality rate of 67.85 per 100,000 compared to 27.65 per 100,000 for White populations (rate ratio 2.45). The Northeast showed more modest disparities (Black: 38.87 vs White: 24.84 per 100,000, rate ratio 1.57), while the South demonstrated intermediate disparities (Black: 46.67 vs White: 25.91 per 100,000, rate ratio 1.80).

## Discussion

This comprehensive analysis of national UGIB mortality data reveals substantial and concerning disparities across racial and geographic dimensions, with implications for emergency endoscopic access and healthcare delivery. The nearly two-fold higher mortality rate among Black/African American populations compared to White populations represents one of the most significant health disparities documented in emergency gastroenterology, demanding immediate attention from healthcare systems, policymakers, and professional societies.

Racial disparities and healthcare access

The observed mortality rate ratio of 1.86 for Black/African American populations cannot be explained by disease prevalence alone and likely reflects complex interactions of healthcare access barriers, socioeconomic factors, and systemic healthcare inequities. Emergency endoscopic intervention within 24 hours of presentation is associated with significantly reduced mortality in UGIB [9], yet access to this critical intervention remains geographically and socioeconomically stratified [10].

Several mechanisms may contribute to these disparities. Delayed presentation to healthcare facilities among minority populations due to financial barriers, insurance limitations, or mistrust of healthcare systems may result in presentation with more advanced disease requiring more intensive interventions [11]. Differential access to high-volume centers with 24/7 endoscopic capabilities may limit timely intervention for minority populations who are more likely to receive care at safety-net hospitals with limited resources [12]. Additionally, disparities in recognition and management of UGIB complications by healthcare providers may contribute to differential outcomes [13].

Geographic variations and healthcare infrastructure

The seven-fold variation in mortality rates across states suggests profound differences in healthcare infrastructure, specialist availability, and care delivery systems. States with the highest mortality rates, including Maine and Utah, may face challenges related to rural geography, limited specialist availability, and difficulties maintaining 24/7 emergency endoscopic services [8]. Conversely, states with the lowest mortality rates, such as California and Florida, may benefit from greater urbanization, higher physician density, and more robust emergency care infrastructure.

The regional analysis provides additional insights into healthcare delivery patterns. The West region's lower mortality rates may reflect better integration of emergency endoscopic services, while the Midwest's pronounced racial disparities suggest particular challenges in ensuring equitable access to emergency care for minority populations in this region.

Policy implications for emergency endoscopic access

These findings have direct implications for healthcare policy and resource allocation. The data suggest an urgent need for interventions to improve emergency endoscopic access, particularly in high-mortality regions and for underserved populations. Potential policy interventions include the development of telemedicine and consultation networks connecting rural and underserved hospitals with tertiary centers for emergency endoscopic consultation and patient transfer protocols. Implementation of quality metrics related to time-to-endoscopy and mortality outcomes as part of hospital accreditation and quality reporting requirements represents another critical area for intervention. Targeted recruitment and training programs to increase emergency endoscopy capacity in underserved regions and safety-net hospitals could address workforce shortages. Finally, reimbursement policies that support 24/7 emergency endoscopic services and incentivize quality outcomes rather than volume alone may improve access and outcomes.

Study limitations

Several limitations should be acknowledged. The analysis relies on death certificate data, which may under-ascertain UGIB-related deaths or misclassify cause of death, particularly in cases with multiple comorbidities. The limited representation of minority populations in many states restricts our ability to assess disparities comprehensively across all geographic regions. The ecological nature of the analysis precludes examination of individual-level factors such as insurance status, comorbidities, or specific treatment received.

The study period encompasses the COVID-19 pandemic (2020-2023), which may have affected healthcare utilization patterns and mortality rates in ways not captured by this analysis. Finally, the analysis cannot directly measure access to emergency endoscopic services, limiting our ability to establish causal relationships between observed disparities and healthcare access barriers.

Clinical implications

For clinicians and healthcare systems, these findings underscore the importance of recognizing and addressing disparities in UGIB care. Emergency departments and gastroenterology services should implement systematic approaches to ensure equitable access to timely endoscopic intervention, including protocols for rapid triage, consultation, and transfer when appropriate. Quality improvement initiatives should specifically monitor outcomes across racial and geographic groups to identify and address disparities at the local level.

Future research directions

Future research should focus on understanding the mechanisms underlying observed disparities and evaluating interventions to improve emergency endoscopic access. Priority areas include detailed analysis of time-to-endoscopy intervals across demographic groups, assessment of hospital-level factors associated with disparities, evaluation of telemedicine and consultation network interventions, and prospective studies of quality improvement initiatives targeting disparity reduction.

## Conclusions

This analysis reveals substantial racial and geographic disparities in UGIB mortality, with Black/African American populations experiencing nearly twice the mortality risk of White populations and seven-fold variations across states. These disparities likely reflect underlying inequities in emergency endoscopic access and healthcare delivery, demanding urgent attention from healthcare systems, policymakers, and professional societies.

The findings call for immediate action to improve emergency endoscopic access, particularly in high-mortality regions and for underserved populations. Targeted interventions, including telemedicine networks, workforce development, quality metrics, and payment reform, represent promising approaches to reducing these disparities and improving outcomes for all patients with UGIB.

As healthcare systems increasingly focus on equity and value-based care, addressing UGIB mortality disparities represents both a moral imperative and an opportunity to demonstrate meaningful progress toward health equity. The substantial variations documented in this analysis provide a clear roadmap for prioritizing interventions and measuring progress toward the goal of equitable emergency gastrointestinal care for all Americans.
